# Predicting the prognosis of hepatocellular carcinoma with the treatment of transcatheter arterial chemoembolization combined with microwave ablation using pretreatment MR imaging texture features

**DOI:** 10.1007/s00261-020-02891-y

**Published:** 2021-01-01

**Authors:** Jun Liu, Yigang Pei, Yu Zhang, Yifan Wu, Fuquan Liu, Shanzhi Gu

**Affiliations:** 1 Department of Interventional Therapy, Beijing Shijitan Hospital, Affiliated Hospital of Capital Medical University, Beijing, 100038 People’s Republic of China; 2 Department of Radiology, Xiangya Hospital, Central South University, Changsha, 410008 Hunan People’s Republic of China; 3 Xiangya Hospital, Central South University, Changsha, 410008 Hunan People’s Republic of China; 4 Department of Interventional Therapy, Hunan Cancer Hospital and the Affiliated Cancer Hospital of Xiangya School of Medicine, Central South University, Changsha, 410006 Hunan People’s Republic of China

**Keywords:** Hepatocellular carcinoma, Transarterial chemoembolization, Microwave ablation, Texture analysis, Prognosis

## Abstract

**Objective:**

To investigate the prognostic value of baseline magnetic resonance imaging (MRI) texture analysis of hepatocellular carcinoma (HCC) treated with transcatheter arterial chemoembolization (TACE) and microwave ablation (MWA).

**Methods:**

MRI was performed on 102 patients with HCC before receiving TACE combined with MWA in this retrospective study. The best 10 texture features were screened as a feature group for each MRI sequence by MaZda software using mutual information coefficient (MI), nonlinear discriminant analysis (NDA) and other methods. The optimal feature group with the lowest misdiagnosis rate was achieved on one MRI sequence between two groups dichotomized by 3-year survival, which was used to optimize the significant texture features with the optimal cutoff values. The Cox proportional hazards model was generated for the significant texture features and clinical variables to determine the independent predictors of overall survival (OS). The predictive performance of the model was further evaluated by the area under the ROC curve (AUC). Kaplan–Meier and log-rank tests were performed for disease-free survival (DFS) and Local recurrence-free survival (LRFS).

**Results:**

The optimal feature group with the lowest misdiagnosis rate of 8.82% was obtained on T2WI using MI combined with NDA feature analysis. For Cox proportional hazards regression models, the independent prognostic factors associated with OS were albumin (*P* = 0.047), BCLC stage (*P* = 0.001), Correlat_(1,− 1)T2_ (*P* = 0.01) and SumEntrp_(3,0)T2_ (*P* = 0.015), and the prediction efficiency of multivariate model is AUC = 0.876, 95%CI = 0.803–0.949. Kaplan–Meier analyses further demonstrated that BCLC (*P* < 0.001), Correlat_(1,− 1)T2_ (*P* = 0.023) and SumEntrp_(3,0)T2_ (*P* < 0.001) were associated with DFS, and BCLC (*P* = 0.007) related to LRFS.

**Conclusions:**

MR imaging texture features may be used to predict the prognosis of HCC treated with TACE combined with MWA.

## Introduction

Hepatocellular carcinoma (HCC) is a highly aggressive malignant tumor, and one of the leading causes of cancer death. It ranks eighth in incidence for women and fifth for men with an annual incidence of more than 660,000 new cases worldwide [1, 2]. Transarterial chemoembolization (TACE) is the first treatment option for patients with unresectable HCC [3, 4]. It is widely accepted as a means to control tumor growth and to prolong survival in patients with unresectable HCCs. But the complete necrosis rate of tumors after TACE is between 10 and 0% [5, 6]. Failure to completely occlude the tumor-supplying artery because of angiogenesis around the residual tumor is the major cause of treatment failure. Hence, combination strategies that use both embolization and ablative methods, such as microwave ablation (MWA), have emerged to improve the clinical outcomes of TACE [7].

Previous studies on HCC have reported that the clinical efficacy of TACE combined with MWA is better than that of either therapy alone [8, 9]. Assessing survival outcomes using clinicopathological data have limited prognostic value due to a lack of detailed quantitative parameters. Identifying reliable quantitative prognostic markers, therefore, remains a difficult but essential goal.

Texture analysis evaluates the heterogeneity of a tumor by quantifying the gray level intensity or position of the pixels in an image [10, 11]. Recent studies showed that texture analysis could offer information on the tumor microenvironment and help predict pathological characteristics, overall survival (OS), and response to therapy [11–13]. Magnetic resonance imaging (MRI) is widely used to detect and characterize liver lesions, and to monitor and predict the treatment response of hepatic tumors [14, 15]. However, to the best of our knowledge, no study has used MRI-based texture analyses to predict prognosis after TACE combined with MWA. The purpose of our study is to assess the value of pre-therapeutic MRI texture analysis in predicting the prognosis of HCC after combination therapy.

## Materials and methods

### Patients

This retrospective single-center study was approved by the Medical Ethics Committee of our institution, and the requirement for informed consent was waived.

**Fig. 1:**
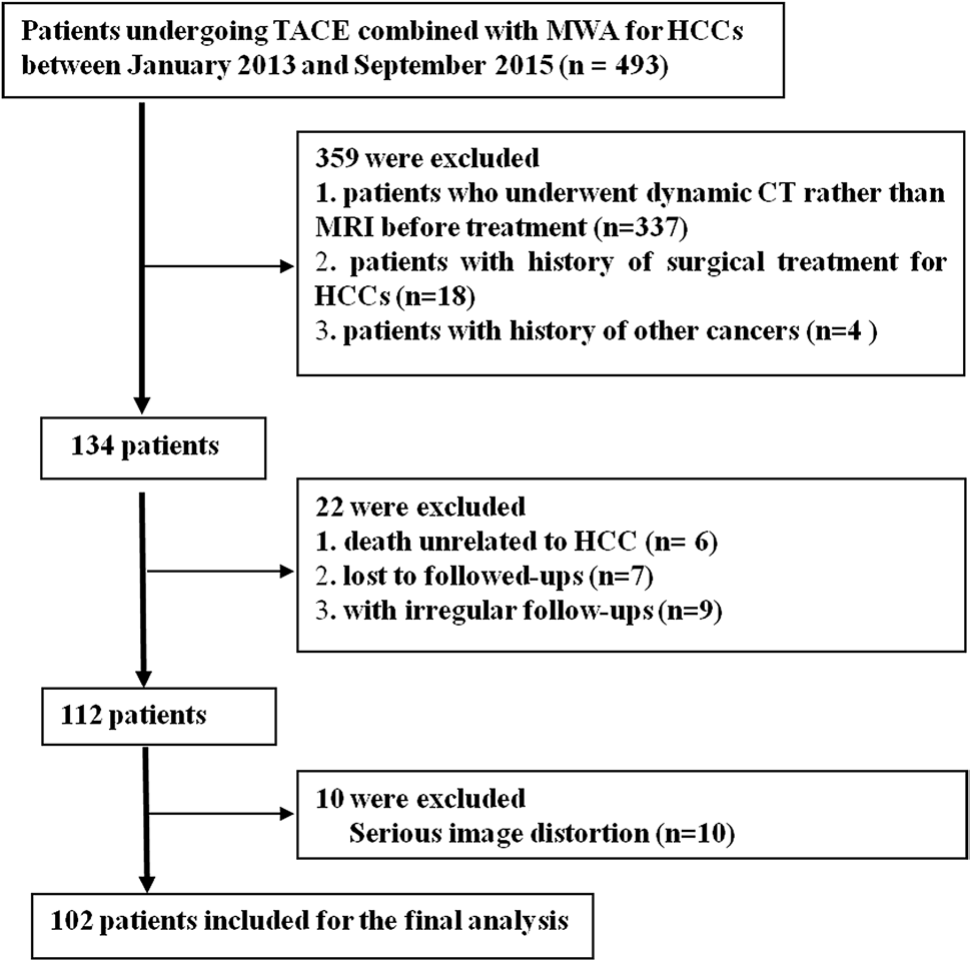
Flowchart for screening HCC patients treated with TACE and MWA in our hospital

The study population consisted of 102 patients diagnosed with HCC according to the American Association for the Study of Liver Disease between January 1, 2013, and September 1, 2018. All patients were treated with TACE combined with MWA in our hospital (Fig. 1). All included Patients' characteristics are shown in Table 1. The inclusion criteria were as follows: (1) no previous treatment; (2) Barcelona Clinic Liver Cancer (BCLC) stage: 0, A, or B; (3) postoperative survival > 2 months; and (4) received TACE and MWA at our institution. Exclusion criteria were as follows: (1) initially diagnosed with CT, not MRI; (2) HCC after surgical treatment; (3) with history of other cancers; (4) death unrelated to HCC; (5) Lost to followed-ups; (6) with irregular follow-ups, no sufficient data for evaluating OS and prognostic factors; and (7) Serious MR image distortion.

**Table 1:** Characteristics of the patients included in this study

Variables	Number of patients
Gender	
Male	88 (86.3%)
Female	14 (13.7%)
Age (y)	
≤ 60	58 (56.9%)
> 60	44 (43.1%)
BCLC stage	
0	7 (6.9%)
A	64 (62.7%)
B	31 (30.4%)
Child–Pugh class	
A	86 (84.3%)
B	15 (14.7%)
C*	1 (1.0%)
Cause of disease	
HBV	89 (87.3%)
Others^a^	13 (12.7%)
Closing to the extrahepatic organ	
No	42 (41.2%)
Yes	60 (58.8%)
Closing to a large vessel	
No	49 (48.0%)
Yes	53 (52.0%)
Tumor maximum diameter (cm)	
≤ 3	43 (42.2%)
3–5	32 (31.4%)
≥ 5	27 (26.5%)
Lesion number (*N*)	
*N* = 1	78 (76.5%)
*N* > 1^b^	24 (23.5%)
TBIL (μmol/L)	
≤ 20	70 (68.6%)
> 20	32 (31.4%)
ALB (g/L)	
≤ 35	35 (34.3%)
> 35	67 (65.7%)
ALT (U/L)	
≤ 40	65 (63.7%)
> 40	37 (36.3%)
ALP (U/L)	
≤ 65	48 (47.1%)
> 65	54 (52.9%)
GGT(U/L)	
≤ 50	42 (41.2%)
> 50	60 (58.8%)
PT(s)	
≤ 13	43 (42.2%)
> 13	59 (57.8%)
AFP(ng/mL)	
≤ 20	42 (41.2%)
20 ~ 200	32 (31.4%)
≥ 200	28 (27.5%)

*AFP* Alpha fetoprotein level, *ALT* alanine aminotransferase, *TBIL* total bilirubin, *GGT* glutamyl transferase, *ALB* albumin, *ALP* alkaline phosphatase, *PT* prothrombin time

*After protecting the liver and relieving jaundice, Child–Pugh C class was degraded to Child–Pugh B class, and the patient can tolerate the following treatment

^a^Including 1 case of HCV, 1 case of cirrhosis due to schistosomiasis, 1 case of Budd–Chiari syndrome, 2 case of alcoholic cirrhosis, and 8 cases of unknown cause

^b^Including 18 cases of two nodules, 6 cases of three nodules

### Candidate clinical factors

We chose the following clinical features for the Cox proportional hazard models: age, sex, hepatitis B viral infection (or other serotypes); Barcelona Clinic Liver Cancer (BCLC) stage (0, A, or B); Child−Pugh class (A, B, or C); maximum diameter (MD) of the lesion, number of lesions (1, or > 1); proximity to a large vessel (yes or no: yes = tumor margin is less than 5 mm from the portal vein, hepatic vein or inferior vena cava and their branches (larger than 3 mm in diameter) or no = tumor margin is more than 5 mm from the large vessels); proximity to extrahepatic organs (yes or no: yes = tumor margin is less than 5 mm from the gastrointestinal tract, liver capsule, diaphragm, and kidney or no = tumor margin is more than 5 mm extrahepatic organs); Alpha-fetoprotein level (AFP ≤ 20 ng/mL, 20–200 ng/mL or ≥ 200 ng/mL); alanine aminotransferase (ALT ≤ 40U/L or > 40U/L); total bilirubin (TBIL ≤ 20 μmol/L or > 20 mol/L); glutamyl transferase (GGT ≤ 50 U/L or > 50 U/L); albumin (ALB ≤ 35 g/L or > 35 g/L); alkaline phosphatase (ALP ≤ 65 U/L or > 65 U/L) and prothrombin time (PT ≤ 13/s or > 13/s).

### Therapy procedure

TACE was performed within 2 weeks after the diagnosis of HCC. Patients were infused with lobaplatin (50 mg/m^2^), and then iodized oil emulsion mixed with epirubicin (30 mg/m^2^); a microcatheter was then inserted into the tumor feeding artery. If necessary, gelatin sponge particles (150–350 μm) were injected until the flow was static. Liver and kidney functions were evaluated after TACE to ensure safe MWA. CT-guided MWA was sequentially performed at approximately 7 days after TACE. One or two 14 G antennae were inserted deep into the target lesion. The microwave power was set at 60–80 W, and the procedure lasted 10–20 min. For tumors with clear boundaries, the ablative volume enveloped the entire tumor including a 0.5–1.0 cm margin surrounding normal tissue. For tumors with irregular shapes or with obscure boundary, the ablative volume enveloped the entire tumor with a margin of 1.0 cm or more. Multiple overlapping ablations were used for tumors > 3.5 cm. For irregular tumors larger than 5.0 cm, enhanced CT within 3–7 days after the treatment was used to detect any residual viable tissue that would require the second MWA. Vital signs such as blood pressure, heart rate, and oxygen saturation were monitored during the procedure. Hepatoprotective, anti-inflammatory, analgesic, and symptomatic treatment were prescribed after MWA.

### Follow-up

The patients were followed up by telephone or clinical visits 4 weeks after MWA and then every 3 months. Physical examination, hepatic function tests, AFP level, and triphasic contrast-enhanced CT or MRI were reviewed. The decision was made regarding treatment response, evidence from current guidelines, and the patients' status and intention to treat. For patients with tumor recurrence, an effective treatment plan was determined by our multidisciplinary team (MDT). Tumor recurrence included local and intrahepatic recurrence. Local tumor recurrence was defined as the presence of enhancement within or around the treated area, and intrahepatic recurrence was the presence of enhancement outside the treated area of the tumors > 1 month after treatment. OS, local recurrence-free survival (LRFS), and disease-free survival (DFS) were also assessed. All recurrences were confirmed by CT or MRI. OS was defined as the time from baseline MRI to death or end date. LRFS was defined as the time from TACE to local recurrence, death, or end date. DFS was defined as the time from TACE to local and intrahepatic recurrence, death, or end date. Patients were followed up until death or September 1, 2018, if they were still alive.

### MR imaging protocol

The pre-therapeutic MR imaging was performed with a 3.0-T scanner (Achieva; Philips Medical Systems, Best, the Netherlands) with a 16-channel dedicated phased-array body coil. The abdominal MR protocol consisted of the following sequences: (1) axial T2-weighted fat-suppressed 2D turbo-spin-echo (TSE); repetition time (TR)/echo time (TE), 3000/70 ms; slice thickness, 5 mm; slice gap, 1.1 mm; matrix, 320 × 280; (2) axial T1-weighted and contrast-enhanced imaging: T1WI three-dimensional turbo field echo sequence (T1 high-resolution isotropic volume examination, THRIVE, Philips Healthcare) was performed before and after injection of gadopentetate dimeglumine (Magnevist; Bayer Healthcare, Germany, 0.1 mmol/kg) at a rate of 2 ml/sec followed by a 20-ml saline flush with the following parameters: TR/TE: 4.1/1.4 ms, slice thickness: 1 mm, no slice gap, matrix: 252 × 198, hepatic arterial phase (HAP), portal venous phase (PVP), and equilibrium phase images were obtained at 20–30 s, 70–80 s, and 180 s after contrast medium injection, respectively.

For enhanced MR imaging, only PVP images were selected and reconstructed to obtain texture features [16]. Thus, in our study, contrast-enhanced T1-weighted PVP images reconstructed with 3 mm thickness, T1-weighted images and T2-weighted images, were transferred to personal computers for texture analyses.

### Texture features extraction and selection

**Fig. 2:**
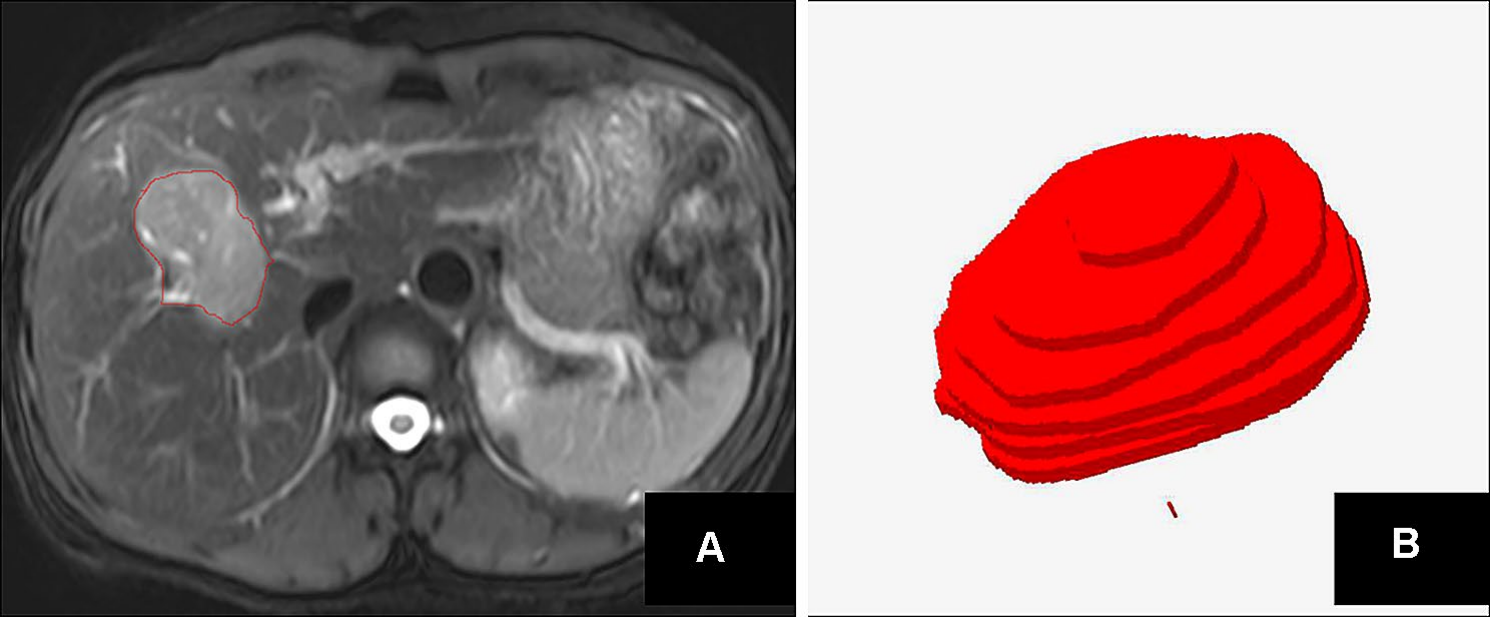
An example of ROI segmentation and VOI generation on T2WI. **a** Shows that the 2D region of interest (ROI) was delineated manually on a T2W image. **b** Presented that 3D view was generated automatically based on all 2D ROIs of the tumor

All the BMP format images, including T1-weighted images (T1WI), T2-weighted images (T2WI) and contrast-enhanced T1WI in portal venous phase (PVP), were transferred into the MaZda program (http://www.eletel.p.lodz.pl/programy/mazda/index.php?action = mazda) for texture analysis. The region of interest (ROI) on the MR parametric maps cannot be utilized automatically by MaZda. Thus, one radiologist (J. L, with 11 years of experience in MRI)—blinded to the clinical and pathological findings—manually traced the tumor border on each axial map, to obtain the corresponding two-dimensional (2D) ROI for each map. Both the most superior and the most inferior slices for each tumor were excluded to avoid volume averaging. Based on all the ROIs from the tumor, a three-dimensional (3D) volume of interest (VOI) was generated automatically (Fig. 2). For each VOI, a total of 229 texture features was extracted automatically by MaZda [17]. Nine first-order texture features were described by the histogram of the signal intensity values of pixels in the VOI. 220 s-order texture features (gray level co-occurrence matrix features, GLCM) were derived from 20 co-occurrence matrices produced from 4 directions and 5 inter-pixel distances in the VOI (Table 2).

**Table 2:** Information about texture features

Feature category	Name	Description
First-order texture features	Mean	Average intensity across the VOI
	Variance	Dispersion from the mean value
	Skewedness	Asymmetry
	Kurtosis	Peakedness or pointedness
	Perc1%, Perc10%, Perc50%, Perc90% and Perc99%	MR number percentiles indicate the attenuation value below which each percentage of voxels in the respective VOI area lie
GLCM features	Entropy	Measure of the randomness of the gray levels
	AngScMom (angular second moment)	Measure of image homogeneity
	DifEntrp (difference entropy)	Measure of the randomness of the difference of neighboring voxels'gray levels
	Difvarnc (difference variance)	Measure of variations of difference of gray levels between voxel pairs
	InvDfMom (inverse difference moment)	Measure of the image homogeneity
	SumAverg (sum average)	Measure of the overall image brightness
	SumEntrp (sum entropy)	Measure of the randomness of the sum of gray levels of neighboring voxels
	SumOfSqs (sum of squares)	Measure of the spread in the gray level distribution
	SumVarnc (sum variance)	Measure of the spread in the sum of the gray levels of voxel pairs distribution
	Contrast	Measure of local image variations
	Correlat	Measure of image linearity

**Fig. 3:**
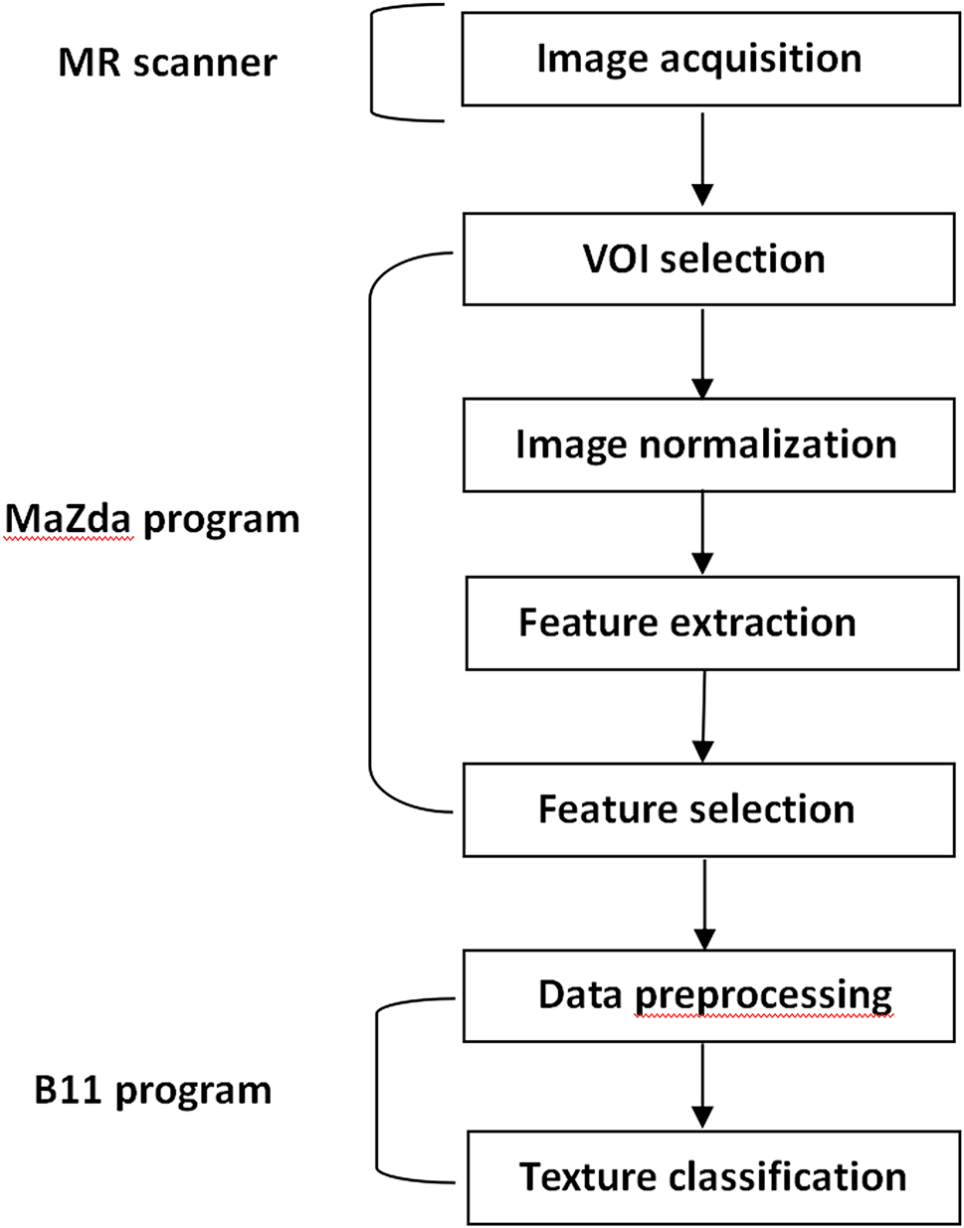
Main steps of MR image texture analysis

The discriminant analysis was done with the Mazda software. The best 10 texture features that predicted 3-year survival were screened out using the following statistical methods: Fisher coefficient, classification error probability with average correlation coefficients (POE + ACC), and mutual information coefficient (MI), respectively. Then texture classification was done using the B11 module in the Mazda software. Three different methods, including the principal component analysis (PCA), linear discriminant analysis (LDA), and nonlinear discriminant analysis (NDA), were applied to calculate the error rate for predicting 3-year survival (Fig. 3). The optimal feature group with the lowest misdiagnosis rate was obtained on one MRI sequence and was used for further analysis.

### Statistical analysis

SPSS, version 20.0 (IBM SPSS, Armonk, NY, USA) and the R software, version 3.5.1 (R Foundation for Statistical Computing, Vienna, Austria) were used for statistical analyses. *P* < 0.05 was regarded as statistically significant. The differences in the values of texture features in the optimal feature group dichotomized by 3-year survival were investigated using independent-sample *t*-tests. The receiver operating characteristic (ROC) was used to explore the diagnostic performance of these identified texture parameters by independent-sample *t*-tests, and to determine the cutoff value that would yield the best sensitivity and specificity to predict 3-year survival. Univariate analysis was performed using the Kaplan–Meier method and log-rank test concerning 16 clinical factors affecting survival.

The texture features with statistical significance were divided into two groups based on the cutoff value. These significant texture features, and other significant clinical factors (screened by univariate analyses), were entered into a multivariate regression analysis by Cox proportional hazards model to predict OS (Method: forward LR; probability for stepwise: entry variables ≤ 0.05, removal variables > 0.1). To further identify the predictive performance of the multivariate Cox regression models for OS, we applied the area under the ROC curve (AUC). Survival curves for LRFS and DFS were obtained using the Kaplan–Meier method and log-rank tests.

## Results

Among the 102 patients, 63 (61.8%) had tumor recurrence (8 local tumor recurrence and 55 intrahepatic recurrences), and 29 (28.4%) died by the end of the follow-up date. The median survival time was 39 months (range 2–67). Three-year OS rate was 73.5% (75/102), and the 3-year recurrence rate was 53.9% (55/102).

With regard to clinical factors, univariate analyses included: age (*P* = 0.590), sex (*P* = 0.661), type of viral hepatitis (*P* = 0.693), BCLC stage (*P* < 0.001), Child–Pugh class (*P* = 0.037), maximum tumor diameter (*P* < 0.001), lesion number (*P* = 0.119), proximity to a large vessel (*P* = 0.019), proximity to an extrahepatic organ (*P* = 0.088), AFP (*P* = 0.369), ALT (*P* = 0.457), total bilirubin (*P* = 0.065), GGT (*P* = 0.205), ALB (*P* = 0.018), ALP (*P* = 0.107), and PT (*P* = 0.269). Subsequently, BCLC stage, Child–Pugh class, maximum tumor diameter, proximity to a large vessel, and albumin (all *P* < 0.05) were entered into Cox proportion hazards models.

The best 10 texture features were obtained for each MRI sequence, and the optimal feature group with the best predictive performance was obtained on T2WI. MI combined with NDA feature analysis, showed that the group with optimal features has the lowest misdiagnosis rate (8.82%) (Table 3).

**Table 3:** Comparing the minimum misdiagnosis rate in three MR sequences using the selective feature methods combined with different feature analysis methods between two groups dichotomized by 3-year survival

	PCA (*n *(%))	LDA (*n *(%))	NDA (*n *(%))
T1WI			
FiSher	18 (17.64%)	32 (31.37%)	16 (15.69%)
MI	31 (30.39%)	21 (20.59%)	21 (20.59%)
POE + ACC	33 (32.35%)	20 (19.60%)	15 (14.70%)
T2WI			
FiSher	32 (31.37%)	31 (30.39%)	13 (12.75%)
MI	34 (33.33%)	31 (30.39%)	**9 (8.82%)**^**§**^
POE + ACC	33 (32.35%)	21 (20.59%)	16 (15.69%)
T1WI enhanced			
FiSher	35 (34.31%)	34 (33.33%)	21 (20.59%)
MI	23 (22.54%)	17 (16.66%)	12 (11.76%)
POE + ACC	15 (14.70%)	30 (29.41%)	11 (10.78%)

*n* = the case of misdiagnosis, % = the minimum misdiagnosis rate, *FiSher* = Fisher coefficient, POE + ACC = classification error probability combined with average correlation coefficients

*MI* mutual information coefficient, *PCA* principal component analysis, *LDA* linear discriminant analysis, *NDA* nonlinear discriminant analysis

^**§**^The lowest misdiagnosis rate

In the optimal feature group, 9 out of 10 texture features showed a distinct difference (*P* < 0.05) between the two groups dichotomized by 3-year survival. For these significant texture features, the AUC was 0.672–0.760 with univariate analysis for predicting the 3-year survival (Table 4). For the results of Cox proportional hazards regression models, the independent prognostic factors associated with OS were albumin, BCLC stage, Correlat_(1,− 1)_, SumEntrp_(3,0)_, with an AUC value of 0.876, 95% CI = 0.803–0.949 (Table 5). For the Cox proportional hazards regression models, a polynomial equation is used as follow: *h*(*X*,*t*) = *h*0(*t*)exp(1.38*x*^1^ + 0.774*x*^2^ + 1.080*x*^3^ + 1.009*x*^4^). *x*^1^,*x*^2^,*x*^3^,*x*^4^ represent respectively the BCLC stage, albumin ≤ 35 g/L, Correlat_(1,− 1)_ ≥ 0.978, SumEntrp_(3,0)_ ≥ 1.282.

**Table 4:** Differences in the optimal texture features on T2W imaging between two groups dichotomized by 3-year survival

Texture features on T2WI	≥ 3 years	< 3 years	*P* value	Cutoff value	Sensitivity	Specificity	AUC (95%CI)
SumEntrp(2,0)	1.206 ± 0.123	1.319 ± 0.175	0.004*	1.250	70.37%	72.00%	0.709 (0.610–0.794)
Correlat(1,− 1)	0.933 ± 0.049	0.956 ± 0.036	0.032*	0.978	44.44%	94.67%	0.672 (0.572–0.762)
Entropy(3,0)	1.441 ± 0.173	1.595 ± 0.250	0.006*	1.619	51.85%	86.67%	0.702 (0.603–0.788)
SumEntrp(3,0)	1.108 ± 0.136	1.245 ± 0.194	0.002*	1.282	51.85%	89.33%	0.720 (0.622–0.804)
Entropy(4,0)	1.408 ± 0.177	1.589 ± 0.238	0.001*	1.622	51.85%	90.67%	0.729 (0.632–0.812)
SumEntrp(4,0)	1.054 ± 0.141	1.211 ± 0.185	< 0.001*	1.075	77.78%	65.33%	0.753 (0.657–0.833)
SumEntrp(0,4)	1.075 ± 0.186	1.241 ± 0.172	< 0.001*	1.222	66.6%	78.6%	0.760 (0.665–0.839)
Entropy(5,0)	1.370 ± 0.189	1.564 ± 0.247	< 0.001*	1.531	55.56%	82.67%	0.724 (0.627–0.808)
SumEntrp(5,0)	1.000 ± 0.160	1.162 ± 0.201	< 0.001*	1.105	62.96%	78.67%	0.736 (0.640–0.819)
SumAverg(2,0)	49.642 ± 10.517	49.230 ± 6.323	0.811	–	–	–	–

Texture features = the optimal feature group. The values of texture features = the mean value ± standard deviation for texture features. The number in the parenthesis for texture features on T2WI represented the coordinate of the matrix

*AUC* area under the curve, *CI* confidence interval

^*^Statistical significance

**Table 5:** Multivariate analysis for overall survival with Cox proportional hazards model

Parameter	B	SE	*P* value	Wald	HR (95%CI)
BCLC stage	1.380	0.404	0.001	11.648	3.977(1.800–8.786)
ALB	0.774	0.389	0.047	3.962	2.168(1.012–4.646)
Correlat (1,− 1)	1.080	0.420	0.010	6.611	2.944(1.293–6.705)
SumEntrp (3,0)	1.009	0.413	0.015	5.951	2.742(1.219–6.166)

The number in the parenthesis represented the coordinate of matrix in various second-order texture features

*BCLC* Barcelona Clinic Liver Cancer, *ALB* albumin, *B* partial regression coefficient, *SE* standard error, *Wald* = Wald coefficient, *HR* = hazard ratio, *CI* confidence interval

For albumin; using the Kaplan–Meier method and log-rank tests, the negative group (*n* = 67; 14 deaths and 38 recurrences (5 local recurrences)) and the positive group (*n* = 35; 15deaths and 25 recurrences (3 local recurrences)) had no statistically significant difference in DFS (*χ*^2^ = 2.286; *P* = 0.131) and LRFS (*χ*^2^ = 0.057; *P* = 0.811).

**Fig. 4:**
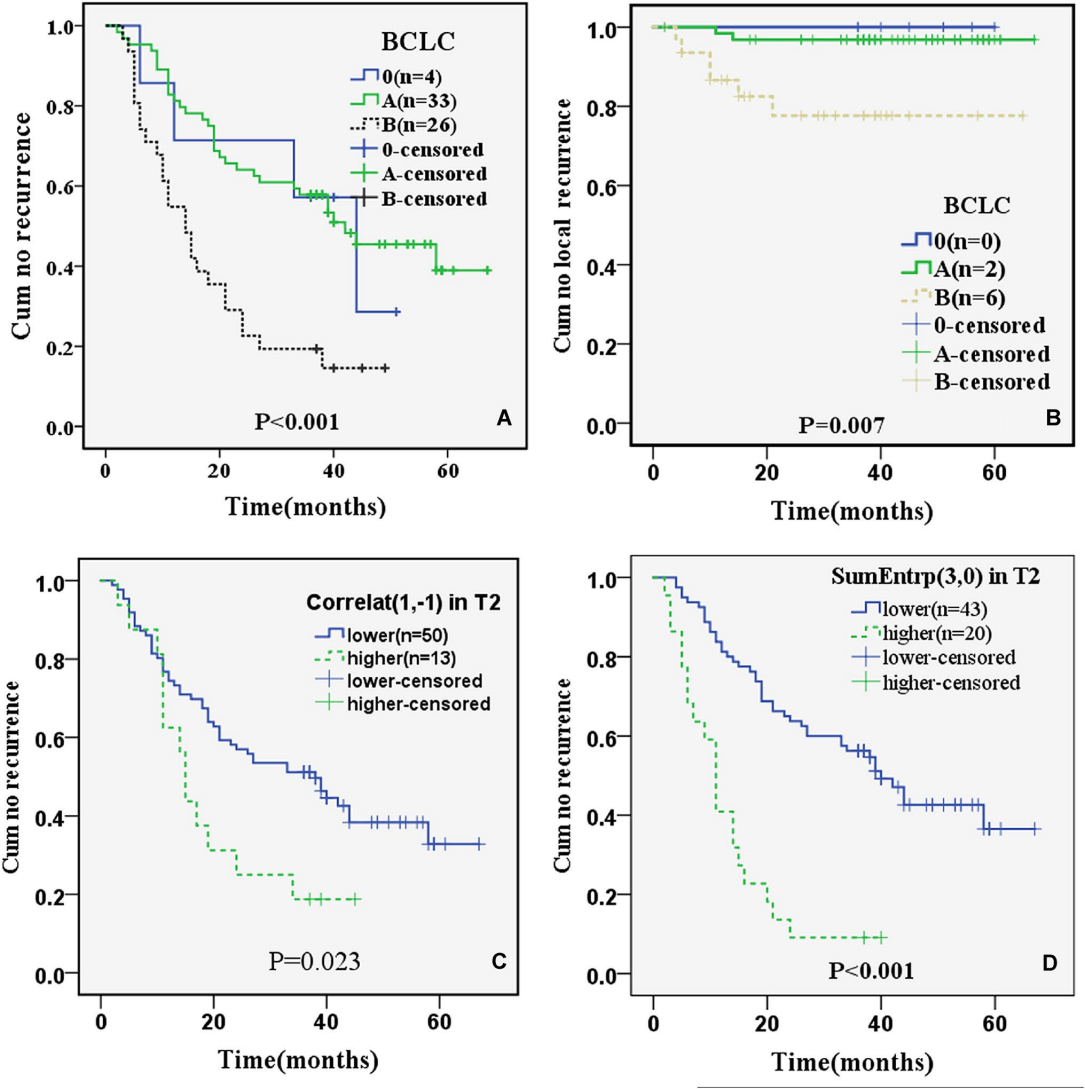
Kaplan–Meier survival curves for DFS separated by BCLC (*P* < 0.001) (**a**), Correlat _(1,− 1)_ (*P* = 0.023) (**c**), SumEntrp _(3,0)_ (*P* < 0.001) (**d**), and for LRFS separated by BCLC (*P* = 0.007) (**b**). Note. The number in the parentheses was the patients with HCC recurrence (**a**, **c**, **d**) or local recurrence (**b**) in during the follow-up Period

For BCLC; stage 0 (*n* = 7; 0 deaths and 4 recurrences (0 local recurrences)), stage A (*n* = 64; 11 deaths and 33 recurrences (2 local recurrences)), stage B (*n* = 31; 18 deaths and 26 recurrences (6 local recurrences)) had statistically significant differences in DFS (*χ*^2^ = 17.980; *P* < 0.001) (Fig. 4a) and LRFS (*χ*^2^ = 10.038; *P* = 0.007) (Fig. 4b).

For T2WI Correlat_(1,− 1)_ (range: 0.737–0.996) divided by the threshold of 0.978; the lower group (*n* = 86; 17 deaths and 50 recurrences (5 local recurrences)) and the higher group (*n* = 16; 12 deaths and 13 recurrences (3 local recurrences)) had significant differences in DFS (*χ*^2^ = 5.136; *P* = 0.023) (Fig. 4c), but no significant difference in LRFS (*χ*^2^ = 3.473; *P* = 0.062).

For T2WI SumEntrp_(3,0)_ (range: 0.709–1.601) divided by the threshold of 1.282, the lower group (*n* = 80; 14 deaths and 43 recurrences (6 local recurrences)) and the higher group (*n* = 22; 15 deaths and 20 recurrences (2 local recurrences)) had statistical differences in DFS (*χ*^2^ = 29.575; *P* < 0.001) (Fig. 4d), but no significant difference in LRFS (*χ*^2^ = 0.192; *P* = 0.661).

Therefore, three parameters (BCLC stage, Correlat_(1,− 1)_ and SumEntrp_(3,0)_ on T2WI) were significantly associated with DFS. In addition, one BCLC stage was significantly associated with LRFS.

## Discussion

TACE sequentially combined with MWA has been widely accepted as an effective treatment option for surgically unresectable HCC. To evaluate the prognosis of HCC after this combination treatment, we collected clinical data and pre-therapeutic MRI texture features of 102 patients. In our study, texture features obtained from T2W images had the least misdiagnosis rate of 8.82%, and therefore, had the best performance in predicting 3-year survival. This result is consistent with a previous study [18]. T2WI has a dynamic range of images with the lowest signal intensity being 0 and the highest determined by bile. Since the highest and lowest signal intensities can always be determined by the above two elements, the calculation of texture features from T2WI may be more reliable than from T1WI and enhanced T1WI.

Medical images provide sufficient information about tumor morphology as well as heterogeneity to enable tumor characterization and prognostication. Texture analysis provides crucial information regarding a tumor by evaluating its heterogeneity [19]. Texture quantification of MR images is useful in characterizing liver lesions, especially the atypical HCC in a non-cirrhotic liver where the diagnosis can be challenging even with the liver-specific contrast-enhanced MRI [20]. The clinical value of texture analysis cannot be overemphasized: it has been used to predict the HCC response after TACE [21], to determine the most appropriate treatment option between liver resection and TACE [22]and prevent unnecessary treatment.

In our research, three texture feature classifications (SumEntrp, Entropy, and Correlat) are vital prognostic factors for HCC treated with TACE and MWA. Our findings are similar to previous studies, which showed that high heterogeneity on MRI maps is usually a sign of poor outcome [23–26]. Kolarevic et al. showed that SumEntrp(Sum Entropy)—a reflection of internal pixel distribution patterns—is a vital measure of tissue heterogeneity [23]. Kim et al. [24] suggested that higher Entropy on T2WI exhibited poorer recurrence-free survival in patients with breast cancer. Also, compared with other conventional prognostic parameters, Entropy appears to be the most robust and strongest independent predictor of 2-year progression-free survival in patients with non-small cell lung cancer [25]. Meyer et al. found that Correlat had the best correlation with Ki67 index (*r* = 0.75), which was the clinically most relevant marker for predicting the proliferative activity of tumors [26].

Texture features are classified into first-, second- and higher-order statistics. The first-order statistics, based on histogram analysis, reflect the intensity distribution of a VOI. The second-order statistics (an example is GLCM), such as SumEntrp, Correlat and Entropy, can reflect the spatial relationship between gray values; and is promising in predicting the therapeutic response and prognosis of many kinds of tumors [27, 28]. For the first-order texture features, previous studies [29, 30] showed that Variance and Skewness indicate high tumor heterogeneity, and predict poor patient prognosis. However, none of them is included in the optimal feature group in our study because GLCM provides more texture information about tumor heterogeneity [31, 32] that is superior to the first-order features in predicting the prognosis of HCC. SumEntrp_(3,0)_ and Correlat_(1,− 1)_ were the independent predictors of OS in patients with HCC after TACE combined with MWA. Therefore, the GLCM features may be as powerful as clinical variables in predicting the prognosis of malignancies including HCC.

The BCLC stage and albumin were independent prognostic factors associated with OS, and the BCLC stage was also associated with DFS. The BCLC staging system is widely used by clinicians because it incorporates multiple variables, including tumor size, hepatic function, and performance status of patients with HCC [33]. Our analysis showed that OS of patients with BCLC stage 0 (*n* = 7) and A (*n* = 64) was longer compared to patients with BCLC stage B (*n* = 31). DFS and LRFS of patients with BCLC stage 0 (*n* = 4 (DFS); *n* = 0 (LRFS)) and A (*n* = 33 (DFS); *n* = 2 (LRFS)) were longer than that of stage B (*n* = 26 (DFS); *n* = 6(LRFS)). Also, the prognosis of HCC is closely related to autoimmunity and the level of inflammatory response. Preoperative ALB reflects liver function status and immune level. Some studies have suggested that the ALB level is an independent factor influencing the prognosis and recurrence of HCC [34]. Therefore, attention should be paid to understanding the preoperative ALB in predicting the prognosis of HCC.

Our study had several limitations. First, it was a retrospective study prone to potential selection bias. Second, we have a small sample size; 337 of 493 patients were excluded because they had dynamic CT rather than MRI before TACE combined with MWA. Third, we used a thick slice reconstruction in our study, however, the variation in the slice thickness of MR images doesn't significantly affect the robustness of texture features [35, 36]. Further studies with larger patient populations, enhanced MRI features, and molecular biological indicators are needed to analyze the relevance between texture features and prognosis for HCC.

In conclusion, our study did not impose extra burdens on patients, as we used routinely acquired MR images. Furthermore, there are four parameters associated with OS, three associated with DFS, and one associated with LRFS. The present study showed that the 3D texture features based on MRI might be useful in predicting the prognosis of HCC before starting TACE combined with MWA.

## Abbreviations

HCC: Hepatocellular carcinomas
TACE: Transcatheter arterial chemoembolization
MWA: Microwave ablation
ROC: Receiver operating characteristic
DFS: Disease-free survival
OS: Overall survival
ROI: Region of interest
GLCM: Gray level co-occurrence matrix
PVP: Portal venous phase

## References

[1] Ferenci P, Fried M, Labrecque D, et al. World Gastroenterology Organisation Guideline. Hepatocellular carcinoma (HCC): a global perspective. J Gastrointestin Liver Dis. 2010;19(3):311–7. 20922197

[2] Venook AP, Papandreou C, Furuse J, de Guevara LL (2010). The incidence and epidemiology of hepatocellular carcinoma: a global and regional perspective. Oncologist.

[3] Padhya KT, Marrero JA, Singal AG (2013). Recent advances in the treatment of hepatocellular carcinoma. Curr Opin Gastroenterol.

[4] McCurdy HM (2013). Improving outcomes for patients receiving transarterial chemoembolization for hepatocellular carcinoma. Gastroenterol Nurs.

[5] Kim KM, Kim JH, Park IS (2009). Reappraisal of repeated transarterial chemoembolization in the treatment of hepatocellular carcinoma with portal vein invasion. J Gastroenterol Hepatol.

[6] Marelli L, Shusang V, Buscombe JR (2009). Transarterial injection of (131)I-lipiodol, compared with chemoembolization, in the treatment of unresectable hepatocellular cancer. J Nucl Med.

[7] Zhu AX, Abou-Alfa GK (2008). Expanding the treatment options for hepatocellular carcinoma: combining transarterial chemoembolization with radiofrequency ablation. JAMA..

[8] Liu C, Liang P, Liu F (2011). MWA combined with TACE as a combined therapy for unresectable large-sized hepotocellular carcinoma. Int J Hyperthermia..

[9] Xu LF, Sun HL, Chen YT (2013). Large primary hepatocellular carcinoma: transarterial chemoembolization monotherapy versus combined transarterial chemoembolization-percutaneous microwave coagulation therapy. J Gastroenterol Hepatol..

[10] Davnall F, Yip CS, Ljungqvist G (2012). Assessment of tumor heterogeneity: an emerging imaging tool for clinical practice?. Insights Imaging..

[11] Ng F, Ganeshan B, Kozarski R, Miles KA, Goh V (2013). Assessment of primary colorectal cancer heterogeneity by using whole-tumor texture analysis: contrast-enhanced CT texture as a biomarker of 5-year survival. Radiology..

[12] Ganeshan B, Panayiotou E, Burnand K, Dizdarevic S, Miles K (2012). Tumour heterogeneity in non-small cell lung carcinoma assessed by CT texture analysis: a potential marker of survival. Eur Radiol..

[13] Lubner MG, Stabo N, Lubner SJ (2015). CT textural analysis of hepatic metastatic colorectal cancer: pre-treatment tumor heterogeneity correlates with pathology and clinical outcomes. Abdom Imaging..

[14] Shenoy-Bhangle A, Baliyan V, Kordbacheh H, Guimaraes AR, Kambadakone A (2017). Diffusion weighted magnetic resonance imaging of liver: Principles, clinical applications and recent updates. World J Hepatol..

[15] Gong NJ, Wong CS, Chu YC, Gu J (2013). Treatment response monitoring in patients with gastrointestinal stromal tumor using diffusion-weighted imaging: preliminary results in comparison with positron emission tomography/computed tomography. Nmr Biomed..

[16] Fu S, Chen S, Liang C (2017). Texture analysis of intermediate-advanced hepatocellular carcinoma: prognosis and patients' selection of transcatheter arterial chemoembolization and sorafenib. Oncotarget..

[17] Szczypinski PM, Strzelecki M, Materka A, Klepaczko A (2009). MaZda–a software package for image texture analysis. Comput Methods Programs Biomed..

[18] Kinoshita M, Sakai M, Arita H (2016). Introduction of high throughput magnetic resonance T2-weighted image texture analysis for WHO grade 2 and 3 gliomas. PLOS ONE.

[19] Ganeshan B, Miles KA (2013). Quantifying tumour heterogeneity with CT. Cancer Imaging.

[20] Campos JT, Sirlin CB, Choi JY (2012). Focal hepatic lesions in Gd-EOB-DTPA enhanced MRI: the atlas. Insights Imaging..

[21] Park HJ, Kim JH, Choi SY (2017). Prediction of Therapeutic Response of Hepatocellular Carcinoma to Transcatheter Arterial Chemoembolization Based on Pretherapeutic Dynamic CT and Textural Findings. AJR Am J Roentgenol..

[22] Li M, Fu S, Zhu Y (2016). Computed tomography texture analysis to facilitate therapeutic decision making in hepatocellular carcinoma. Oncotarget.

[23] Kolarevic D, Tomasevic Z, Dzodic R, Kanjer K, Vukosavljevic DN, Radulovic M (2015). Early prognosis of metastasis risk in inflammatory breast cancer by texture analysis of tumour microscopic images. Biomed Microdevices.

[24] Kim JH, Ko ES, Lim Y (2017). Breast Cancer Heterogeneity: MR Imaging Texture Analysis and Survival Outcomes. Radiology..

[25] Win T, Miles KA, Janes SM (2013). Tumor heterogeneity and permeability as measured on the CT component of PET/CT predict survival in patients with non-small cell lung cancer. Clin Cancer Res..

[26] Meyer HJ, Schob S, Hohn AK, Surov A (2017). MRI Texture Analysis Reflects Histopathology Parameters in Thyroid Cancer - A First Preliminary Study. Transl Oncol..

[27] Liu J, Mao Y, Li Z (2016). Use of texture analysis based on contrast-enhanced MRI to predict treatment response to chemoradiotherapy in nasopharyngeal carcinoma. J Magn Reson Imaging..

[28] Thibault G, Tudorica A, Afzal A (2017). DCE-MRI Texture Features for Early Prediction of Breast Cancer Therapy Response. Tomography..

[29] Miles KA, Ganeshan B, Hayball MP (2013). CT texture analysis using the filtration-histogram method: what do the measurements mean?. Cancer Imaging..

[30] Pusiol T, Zorzi MG, Morichetti D, Piscioli I, Scialpi M (2013). Uselessness of radiological differentiation of oncocytoma and renal cell carcinoma in management of small renal masses. World J Urol..

[31] Brynolfsson P, Nilsson D, Henriksson R (2014). ADC texture–an imaging biomarker for high-grade glioma?. Med Phys..

[32] Lubner MG, Smith AD, Sandrasegaran K, Sahani DV, Pickhardt PJ (2017). CT Texture Analysis: Definitions, Applications, Biologic Correlates, and Challenges. Radiographics..

[33] Bruix J, Sherman M (2011). Management of hepatocellular carcinoma: an update. Hepatology..

[34] Kinoshita A, Onoda H, Imai N (2015). The C-reactive protein/albumin ratio, a novel inflammation-based prognostic score, predicts outcomes in patients with hepatocellular carcinoma. Ann Surg Oncol..

[35] Waugh SA, Purdie CA, Jordan LB (2016). Magnetic resonance imaging texture analysis classification of primary breast cancer. Eur Radiol..

[36] Savio SJ, Harrison LC, Luukkaala T (2010). Effect of slice thickness on brain magnetic resonance image texture analysis. Biomed Eng Online..

